# Behavioral Engagement and Activation Model Study (BEAMS): A latent class analysis of adopters and non-adopters of digital health technologies among people with Type 2 diabetes

**DOI:** 10.1093/tbm/ibae034

**Published:** 2024-07-02

**Authors:** John D Piette, Keni C S Lee, Hayden B Bosworth, Diana Isaacs, Christian J Cerrada, Raghu Kainkaryam, Jan Liska, Felix Lee, Adee Kennedy, David Kerr

**Affiliations:** School of Public Health, University of Michigan, Ann Arbor, Michigan, USA; Sanofi, Paris, France; School of Medicine, Duke University, Durham, North Carolina, USA; Cleveland Clinic, Cleveland, Ohio, USA; Evidation, San Mateo, California, USA; Evidation, San Mateo, California, USA; Sanofi, Paris, France; Sanofi, Paris, France; Sanofi, Paris, France; Sutter Center for Health Systems Research, Santa Barbara, California, USA

**Keywords:** digital health technology, Type 2 diabetes, user engagement

## Abstract

Many people with Type 2 diabetes (T2D) who could benefit from digital health technologies (DHTs) are either not using DHTs or do use them, but not for long enough to reach their behavioral or metabolic goals. We aimed to identify subgroups within DHT adopters and non-adopters and describe their unique profiles to better understand the type of tailored support needed to promote effective and sustained DHT use across a diverse T2D population. We conducted latent class analysis of a sample of adults with T2D who responded to an internet survey between December 2021 and March 2022. We describe the clinical and psychological characteristics of DHT adopters and non-adopters, and their attitudes toward DHTs. A total of 633 individuals were characterized as either DHT “Adopters” (*n* = 376 reporting any use of DHT) or “Non-Adopters” (*n* = 257 reporting never using any DHT). Within Adopters, three subgroups were identified: 21% (79/376) were “Self-managing Adopters,” who reported high health activation and self-efficacy for diabetes management, 42% (158/376) were “Activated Adopters with dropout risk,” and 37% (139/376) were “Non-Activated Adopters with dropout risk.” The latter two subgroups reported barriers to using DHTs and lower rates of intended future use. Within Non-Adopters, two subgroups were identified: 31% (79/257) were “Activated Non-Adopters,” and 69% (178/257) were “Non-Adopters with barriers,” and were similarly distinguished by health activation and barriers to using DHTs. Beyond demographic characteristics, psychological, and clinical factors may help identify different subgroups of Adopters and Non-Adopters.

## Teaser

Self-efficacy for diabetes management, health activation, and perceived barriers to use technology may help distinguish between subgroups of adopters and non-adopters of digital health tools to manage Type 2 diabetes.

**Implications**

**Practice**: Practitioners and clinicians should include assessments of self-efficacy, health activation, and perceived barriers when tailoring strategies to promote adoption and use of digital health technologies (DHTs) for Type 2 diabetes management.

**Policy:** Wellness programs should consider different subgroups of potential users who are most likely to use and benefit from DHTs to prioritize technical support and encouragement for those who need it the most.

**Research:** Future research should explore how initial user subgroups may differ across types of DHTs and how psychological characteristics and attitudes may change over time as individuals continue to use these tools across different stages of diabetes management.

## Background

Digital health technologies (DHTs), which include mobile health apps, continuous glucose monitors, and telehealth visits with providers, may help improve health outcomes among people with Type 2 diabetes (T2D) [1, 2]. For DHTs to be successful, adoption and effective engagement are critical for maximizing the potential benefits [3, 4]. But despite the increasing availability and popularity of DHTs, adoption remains limited [5] and many DHT adopters either engage with their DHT intermittently or discontinue use before achieving their behavioral or metabolic goals [6]. One explanation is that because individuals’ motivation for diabetes self-management may change over time, a mismatch in the support strategies that a DHT provides (e.g. tracking) and the current needs of an individual may occur, resulting in low engagement or early abandonment [7]. It is therefore imperative to understand the range of needs and challenges that exist among (i) non-adopters to identify subgroups who may benefit from DHTs but do not use them, as well as (ii) adopters to identify subgroups who may need additional support with DHTs. However, little is known about the profiles of distinct subgroups that exist within DHT adopters and non-adopters, and therefore, the type of tailored support needed to promote effective DHT use across a diverse T2D population.

Age, gender, and digital and health literacy have been identified as critical factors driving disparities in access to information technology (“the digital divide”) [8] and thus may help to explain the lack of DHT adoption and engagement. For example, being younger, female, having health insurance, and having higher income are all correlates of adopting mobile health apps [9–12]. As a corollary, older adults often require additional support compared with younger adults to effectively navigate DHTs [13]. For people with diabetes, disease severity, current health status, and psychosocial factors may all impact the adoption of care interventions delivered via DHTs [14]. Psychological characteristics such as knowledge, beliefs, and confidence about disease self-management and attitudes toward digital health, e.g. perceived usability and value, may also be important predictors of adoption and use [15, 16]. One such psychological construct is health activation, which refers to individuals’ level of engagement in their own health care, as measured by the knowledge, skills, and beliefs needed for disease management. It is hypothesized that those with higher levels of activation are more likely to adhere to self-care behaviors such as self-monitoring, exercise, and follow low-fat diets [16].

While demographic, clinical, and psychological characteristics of DHT adopters and non-adopters have been previously described individually, much less is known about how these factors may be interrelated or clustered together in terms of their impact on DHT adoption and use. For example, we know little about how these factors influence initiation of DHT use, how they impact continued engagement, and importantly, how interventions promoting DHT engagement should be tailored based on these characteristics. Statistical methods such as latent class analysis (LCA) provide a useful approach for exploring underlying heterogeneity in a population by allowing researchers to examine whether and how multiple characteristics co-occur to form profiles [17, 18]. While LCA has been used to explore patterns of diabetes self-management behavior [19] and behavioral risk factors for developing diabetes [18], to our knowledge, it has not been used to understand typologies of people who do and do not use DHTs. The co-occurrence of different factors could represent different profiles of DHT adopters and non-adopters, each of which may need different types of support to use the tools.

## Purpose

The objective of the present study was to identify subgroups within DHT adopters and non-adopters among people with established T2D. Identifying these subgroups is important to address individuals’ unique needs during their disease awareness and management journey and could inform the development of tailored strategies for promoting adoption and engagement of DHT services.

## Methods

We conducted an online survey between December 2021 and March 2022 among adults who self-reported having T2D. Survey respondents were primarily recruited through the Evidation Health app (Evidation Health, Inc., San Mateo, CA) [20], an online health community in the USA that includes more than 4 million individuals residing in 50 states and in 90% of zip codes. Members can connect activity trackers and fitness and health apps to the platform and share self-reported health information and consent to participate in research efforts. Approximately half of participants were recruited from this community (53%) and the remainder of respondents were recruited via Facebook. Surveys included questions about the respondent’s demographics, clinical and psychological characteristics (i.e. health activation and self-efficacy), attitudes toward using DHTs, and preferences for DHT features. Respondents were compensated $10. The study was approved by Solutions IRB (#2021/11/16).

Participants were provided the following list of DHT examples and asked if they had ever used any to manage their T2D:

- Using an app on your smartphone to keep track of carbohydrates and check the glycemic index of different foods.
- Using an app on your tablet to take a course on diabetes and healthy living.
- Using a laptop to have a telehealth visit with a diabetes coach.
- Using a device like a continuous glucose monitor at home that is connected to an app on your smartphone to monitor your blood glucose.
- Syncing your smartwatch such as a Fitbit or Apple Watch to track and increase your physical activity.

Based on their responses, participants were labeled as either (i) Adopters: individuals who have ever used DHTs for T2D management and (ii) Non-Adopters: individuals who have never used DHTs.

Clinical and psychological characteristics theoretically relevant to digital health adoption and engagement [14, 15] along with attitudes toward DHT use, were selected for inclusion in the analysis. Binary indicators (yes = 1, no = 0) to be used in the LCA models were constructed from selected survey responses detailed below:

*Clinical characteristics:* Participants reported whether they were currently taking insulin or other diabetes medications and whether they had been diagnosed with depression, anxiety, or another mental health condition. Participants also self-reported their HbA1c range in the past 3 months; those who reported HbA1c of “6.6% to 7.5%” or 7.5% and above” were categorized as “elevated HbA1c.”

*Health activation:* Participants responded to the 10-item Consumer Health Activation Index (CHAI) to assess health activation. Items ask about individuals’ knowledge, beliefs, and behaviors related to managing their personal health. Higher scores are associated with positive lifestyle factors such as fruit and vegetable consumption and exercise. A cutoff score of ≥80 was used to indicate high health activation as outlined by the authors [21].

*Self-efficacy:* Participants responded to the 8-item Self-Efficacy for Diabetes (SED) scale [22] to assess their confidence in self-managing their diabetes. A cutoff score of ≥70 was used to indicate high self-efficacy based on a median split of scores from the sample.

*Attitudes about DHT use:* A series of items were presented to assess a range of attitudes participants had about DHTs. Attitudes that could potentially be addressed through tailored messaging or additional support were prioritized and were grouped into the following dimensions:

(i) technology barriers to using DHTs (e.g. believing DHTs require too much time to use or set up), any from a list = 1;
(ii) need for supportive features (e.g. training on how to use the technology), any from a list = 1;
(iii) concerns about DHTs (e.g. data privacy and security, cost), any from a list = 1;
(iv) trust with data accuracy: moderate or very much trust = 1;
(v) motivations to use DHTs: any social influence to use DHTs = 1, any desire to improve social or work activities = 1, any desire to improve health = 1);
(vi) previous knowledge about DHTs in general yes = 1.

Supplementary Table 1 further details each of these LCA indicators.

### Statistical analysis

LCA was used to explore underlying, unobserved subgroups (latent classes) of individuals among (i) Adopters and (ii) Non-Adopters, separately. Latent classes are characterized by patterns of observed variables, responses to clinical and psychological survey items in this case, that distinguish between groups of respondents. To examine the number of distinct latent classes identifiable within Adopters and Non-Adopters, a series of LCA models was run iteratively on selected LCA indicators with an increasing number of latent classes (starting with 1 class and up to 5 classes). Models were then compared based on model fit indices. The best fitting model, representing the optimal number of latent classes, was selected based on the lowest Bayesian information criterion (BIC) along with theoretical interpretability as recommended [23]. Age, sex, and race/ethnicity were included as covariates in each LCA model. Individuals were assigned to the class for which they had the highest posterior probability of belonging to, given their pattern of responses. Item-response probabilities were summarized descriptively to compare characteristics and attitudes across the identified latent classes. The analysis was conducted using *poLCA*, an R package for Polytomous Variable LCA (R 3.6.3) [24].

## Results

The overall sample consisted of 633 respondents who completed the survey (age 52 ± 12 (mean ± SD) years with a range from 20 to 82 years, 44% female, 67% White, 14% Black/African American, 13% Hispanic/Latino, and 7% Asian). Characteristics of Adopters and Non-Adopters are shown in Table 1. Compared to Non-Adopters, Adopters were younger [mean (SD) age = 49 (11) vs. 56 (12)], less likely to report being female (35% vs. 56%), and more likely to have a bachelor’s or graduate degree (63% vs. 34%). With respect to clinical characteristics, Adopters were less likely than Non-Adopters to report an HbA1c level over 6.5% (50% vs. 65%), depression (24% vs. 41%), and anxiety (20% vs. 34%). Use of different types of insulin medication was similar between cohorts.

**Table 1:** Demographic and clinical characteristics by cohort

		Overall, *N* = 633	Non-Adopter, *N* = 257	Adopter, *N* = 376
Age	Mean (standard deviation)	51.89 (11.88)	56.19 (11.56)	48.95 (11.19)
	Min–Max	20–82	30–82	20–75
Sex (*n*, %)	Female	277 (43.8)	144 (56.0)	133 (35.4)
	Male	355 (56.1)	112 (43.6)	243 (64.6)
	Prefer not to answer	1 (0.2)	1 (0.4)	
Race and/or ethnicity (*n*, %)	White	422 (66.7)	188 (73.2)	234 (62.2)
	Black, African American, African descent, or from the African diaspora	90 (14.2)	33 (12.8)	57 (15.2)
	Hispanic, Latino, or Spanish	85 (13.4)	29 (11.3)	56 (14.9)
	American Indian or Alaskan Native	17 (2.7)	11 (4.3)	6 (1.6)
	Asian, Native Hawaiian, or Other Pacific Islander	42 (6.6)	8 (3.1)	34 (9.0)
	Another race or ethnicity	3 (0.4)	1 (0.4)	2 (0.5)
	Prefer not to answer	3 (0.4)	1 (0.4)	2 (0.5)
Highest level of education (*n*, %)	Some high school; High school degree or equivalent	84 (13.2)	56 (21.8)	28 (10.9)
	Associate’s degree	80 (12.6)	32 (12.4)	48 (12.8)
	Trade/technical/vocational training	33 (5.2)	21 (8.2)	12 (3.2)
	Some college, no degree	108 (17.1)	57 (22.2)	51 (13.6)
	Bachelor’s degree	189 (29.9)	49 (19.1)	140 (37.2)
	Graduate degree	134 (21.2)	38 (14.8)	96 (25.5)
	Prefer not to answer, Missing	5 (0.8)	4 (1.6)	1 (0.3)
Insulin use (*n*, %)	Basal insulin	84 (13.3)	37 (14.4)	47 (12.5)
	Short-acting insulin	88 (13.9)	32 (12.5)	56 (14.9)
	Premixed insulin	61 (9.6)	27 (10.5)	34 (9.0)
	Fixed ratio combination of Glucagon-like peptide-1 receptor agonist (GLP-1RA) and basal insulin	22 (3.5)	2 (0.8)	20 (5.3)
Self-reported HbA1c range in the past 3 months (*n*, %)	HbA1c 6.6% or greater	355 (56.1)	168 (65.4)	187 (49.7)
	HbA1c 6.5% or below	253 (40.0)	72 (28.0)	181 (48.1)
	Missing	25 (4.0)	17 (6.6)	8 (2.1)
Mental health condition (*n*, %)	Depression	195 (30.8)	105 (40.8)	90 (23.9)
	Anxiety	163 (25.8)	88 (34.2)	75 (19.9)

Except for age, table values represent n and column percentage.

Adopters use of DHTs are shown in Table 2.

**Table 2:** DHTs ever used to manage diabetes among adopters

	*n*, %
Activity monitors (e.g. Fitbit, Apple Watch, and Garmin)	337 (89.6)
Nutrition tracking (e.g. MyFitnessPal and Calorie Counter)	197 (52.4)
Websites with educational information about diabetes (e.g. WebMD)	182 (48.4)
Diabetes apps to track data such as blood glucose (e.g. Glucose Buddy and mySugar)	164 (43.6)
Continuous glucose monitors (e.g. FreeStyle Libre and Dexcom G6)	128 (34.0)
Diabetes education programs delivered online or in apps (e.g. WellDoc BlueStar, Livongo, and One Drop)	120 (31.9)
General weight reduction programs (e.g. Weight Watchers and Noom)	109 (29.0)
Telehealth visits with a diabetes coach	59 (15.7)
Other apps or technologies	12 (3.2)

Table 3 shows fit indices used to select the best-fitting model. BIC, Akaike information criterion (AIC), and Log Likelihood are presented; however, BIC is considered more reliable for model selection and thus more commonly used [25]. Entropy, an indicator of classification accuracy, was also reported. Values closer to 1 are ideal, although there is no agreed minimum cutoff and model selection is not based on entropy [25]. Based on the BIC values, where lower values indicate better fit, the best fitting models were a 2-class solution for the Non-Adopter cohort and 3-class solution for the Adopter cohort.

**Table 3:** Model fit indices for latent class models

Model	BIC	AIC	Log likelihood	Entropy
Non-Adopters				
1 Class	4128.185	4082.047	−2028.024	7.891
2 Class	4093.904	3998.078	−1972.039	7.672
3 Class	4111.258	3965.746	−1941.873	7.553
4 Class	4143.909	3948.710	−1919.355	7.472
Adopters				
1 Class	5425.211	5366.267	−2668.134	7.096
2 Class	5305.035	5147.851	−2533.926	6.787
3 Class	5300.809	5045.385	−2457.693	6.670
4 Class	5413.207	5059.544	−2439.141	6.623

Table 4 illustrates the profile of each latent class based on responses to the relevant set of indicators. Item-response probabilities shown in the table represent the probability of responding “yes” to a given LCA indicator, conditional on class membership. Values approaching one, with a darker shading, represent higher probabilities of saying yes. We compare these values across latent classes in order to summarize the distinctive characteristics of each group.

**Table 4:** Item-response probabilities conditional on class membership

	DHT Non-Adopters		DHT Adopters		
	Non-Adopters with barriers, *N* = 178 (69%)	Activated Non-Adopters, *N* = 79 (31%)	Non-activated Adopters with dropout risk, *N* = 139 (37%)	Activated Adopters with dropout risk, *N* = 158 (42%)	Self-managing Adopters, *N* = 79 (21%)
Has concerns with technology	0.63	0.52	0.65	0.17	0.22
Has technology barriers	0.42	0.22	0.30	0.10	0.30
Needs support to use tech	0.38	0.15	0.27	0.30	0.19
Has mental health condition	0.57	0.28	0.45	0.24	0.27
Motivated by daily activities	0.34	0.30	0.35	0.16	0.97
Motivated by social reasons	0.13	0.11	0.29	0.21	0.75
Motivated by health reasons	0.91	0.90	0.86	0.87	1.00
High diabetes self-efficacy	0.20	0.97	0.30	0.73	0.94
High health activation	0.16	0.85	0.22	0.56	0.94
Currently taking insulin	0.28	0.24	0.28	0.20	0.65
Reports elevated HbA1c	0.71	0.53	0.58	0.51	0.31
Trusts data accuracy	0.45	0.71			
Has knowledge about DHTs	0.36	0.47			
Any perceived health improvement from DHT use			0.96	1.00	1.00
Any perceived benefit from DHTs			0.98	0.98	1.00
Would recommend DHTs			0.70	1.00	1.00
Non-adherence			0.09	0.02	0.00

Among Non-Adopters, the larger of the two classes comprised 69% (178/257) of the sample and was characterized as “Non-Adopters with barriers,” while the smaller class was labeled “Activated Non-Adopters” (31% 79/257). Self-efficacy for diabetes management and health activation were key characteristics that distinguished these two classes of non-adopters. The “Activated Non-Adopters” had a high probability of having high self-efficacy as defined by SED scores (0.97) and high health activation as defined by CHAI (0.85). In contrast, the probability of reporting high rates of either attribute in the “Non-Adopters with barriers” class was markedly lower (0.20, 0.16, respectively). Further, the “Non-Adopters with barriers” class had higher probabilities of self-reported elevated HbA1c, having mental health conditions, and reporting barriers to accessing and navigating DHTs (Table 4). Example barriers included believing that DHTs took too much time to set up and that they personally needed training on how to use them. Notably, “Activated Non-Adopters,” did not have a “particular reason” for not using DHTs and believed they could achieve health goals without them, indicating that not all Non-Adopters are expected to need DHTs to manage their T2D.

Among adopters, three classes were identified: 37% (139/376) were “Non-Activated Adopters with dropout risk,” 42% (158/376) were “Activated Adopters with dropout risk,” and 21% (79/376) were characterized as “Self-managing Adopters.” Self-efficacy for diabetes management and health activation similarly distinguished the three classes. “Self-managing Adopters” had the highest probability of having high SED (0.94) and high CHAI (0.94) of the three classes, followed by “Activated Adopters with dropout risk” (0.73 and 0.56, respectively), with the “Non-Activated Adopters with dropout risk” class having lowest probabilities (0.30 and 0.22, respectively).

With respect to the other indicators, “Non-Activated Adopters with dropout risk” were characterized by high probabilities of reporting technology barriers, concerns with technology, and need for support to use DHTs, which are hypothesized to be risk factors for premature disengagement or “dropout.” “Self-managing Adopters” were unique from the other classes with respect to motivators for using DHTs; they had the highest probabilities of receiving encouragement from friends, family, or clinicians to use DHTs (0.75) and for being motivated by daily activities, including returning to social activities and improving work productivity (0.97). “Self-managing Adopters” could be distinguished from the “Activated Adopters with dropout risk” class who had comparatively poorer clinical profiles, i.e. lower probabilities of elevated HbA1c (0.31 vs. 0.51) along with comorbidities not included as LCA indicators: high blood pressure (0.14 vs. 0.58), high cholesterol (0.10 vs. 0.47), and obesity (0.14 vs. 0.39). Poorer clinical profiles among “Activated Adopters with dropout risk,” coupled with higher probability of need for support, suggest that this class may need additional support for effective DHT use avoiding early disengagement.

Among other items not included as LCA indicators, intention to use DHTs in the next 3 months provided support for the distinctiveness and validity of each class. Among Adopters, the “Non-Activated Adopters with dropout risk” class reported the greatest rate of individuals “not interested” in using DHTs to manage their diabetes in the next 3 months (42%), in contrast to 35% in “Activated Adopters with dropout risk” and 9% in “Self-managing Adopters.” Among Non-Adopters, only 19% of “Non-Adopters with barriers” and 11% of “Activated Non-Adopters” were “not interested,” indicating promise for adoption, potentially if the concerns and barriers outlined are addressed.

## Discussion

Some healthcare decision-makers have raised concerns that digital interventions can exclude people with diabetes who are vulnerable due to social determinants of health. A significant emphasis has been placed on these individuals’ access to cellphones and the Internet, giving rise to the “digital divide” [8, 26]. However, nationally representative data suggest that the digital divide—while still important—is smaller in the USA than it was in the past. As of April 2021, 85% of adults in the USA had a smartphone, as did 83% of Black Americans, 75% of those with at most a high school education, and 76% of adults with annual incomes less than $30 000 [27].

The present study sheds light on the drivers of DHT adoption by adults with T2D beyond sole focus on demographic factors and social determinants of health using a novel approach to explore critical attitudes and beliefs that contribute to engagement with DHT tools. This analysis revealed that health activation and self-efficacy helped to distinguish latent subgroups for both Adopters and Non-Adopters. These classes could then be further examined with respect to the types of barriers and concerns about DHTs that are more prevalent depending on latent class. Individual characteristics such as attitudes toward DHTs and perceived barriers are of particular interest because they can be modified through intervention, while demographic and clinical characteristics cannot.

Diabetes self-care support interventions too often are evaluated without consideration of elements that facilitate implementation in the real world (i.e. reach, resources, or cost) [28]. Findings from the current study may help researchers and DHT solution designers develop an approach for identifying and characterizing individuals who are likely to initiate and continue engagement with DHTs. Among Non-Adopters for instance, these findings suggest that adoption-promoting messages should address perceived barriers prevalent in the low self-efficacy/activation “Non-Adopters with barriers” class (i.e. cost, motivation, and technology concerns). In contrast, messages addressing concerns about data privacy and perceived value concerns should be addressed in the high self-efficacy/activation “Activated Non-Adopters” class.

Attitudes and barriers to technology use that emerged, particularly among the “Non-Adopters with barriers” and “Non-Activated Adopters with dropout risk” classes, are consistent with recent research among older adults highlighting beliefs such as a lack of need for technology, cost concerns, and desire for adequate training as important when forming intentions to use technology for heart failure [29]. Furthermore, presence of a mental health condition was highly prevalent among these two classes relative to the other classes and aligns with previous research highlighting suboptimal diabetes self-care behaviors [30] and low engagement with digital interventions among those with depression [31]. The prevalence of mental health conditions among these two low activation/low self-efficacy classes suggests that support for mental health should be considered both when deciding what mental health resources should be included in DHTs for diabetes management and what additional support is needed after initiation.

Notably, further inspection of items included within the “Has concerns with technology” indicator showed that concerns about data privacy and security were common in both groups, mirroring a recent consumer survey suggesting that concern about privacy and data security was the primary barrier to DHT adoption [32]. More broadly, it has been argued that technology introduced into the healthcare system, including mobile apps, artificial intelligence, and virtual reality, require procedures and guidelines underpinning their development to promote trust among patients, healthcare providers, and medical/public health authorities [33]. These findings underscore the importance of addressing trust in DHTs as critical for encouraging adoption and reducing disengagement. Further research will be required to develop effective trust-promoting strategies.

The two classes with highest health activation and self-efficacy, “Activated Non-Adopters” and “Self-managing Adopters” deserve further discussion. There are likely multiple reasons why Adopters used DHTs despite having similar activation and self-efficacy as Non-Adopters, including more exposure to DHTs, greater personal interest and perceived value, and the possibility that high activation and self-efficacy are a result of DHT use rather than a prerequisite. Notably, we observed that “Self-managing Adopters” had higher probabilities of reporting current insulin use and physician recommendation to use DHTs than “Activated Non-Adopters” and lower probabilities of reporting elevated HbA1c compared to the other classes. It is possible that physician recommendation was the antecedent to DHT use among “Self-managing Adopters,” perhaps in conjunction with recommendations to start insulin. Indeed, a recent systematic review highlighted the role of physician recommendation as an important driver of diabetes management app use [11]. Our findings suggest that encouraging physician recommendation of effective DHTs may be one promising strategy for increasing adoption, particularly among those with high activation and self-efficacy. Finally, it is important to note that not all Non-Adopters, activated or otherwise, need to use DHTs systematically. Recommendation for use may depend on factors not considered in the present analysis, such as individuals’ current mental and emotional attitude and receptivity toward self-care, which may shift over time [34]. However, it is noteworthy that both classes in the Non-Adopter cohort contain high probabilities of having elevated HbA1c and thus DHT use may be explored for those individuals.

Limitations should be considered when interpreting the findings from this study. Regarding selection bias, Non-Adopters were recruited via digital platforms and likely underrepresent individuals who use technology (e.g. internet, social media, etc.) less frequently or not at all. We argue, however, that internet and social media users are likely the target population for adoption and engagement strategies. The relatively small population of the USA with suboptimal or no access to the internet may require additional support to use DHTs beyond the scope of our discussion. LCA is a probabilistic model, such that individuals are assigned to the cluster in which they have the highest probability of membership; there may be a subset of individuals who have similar probabilities for membership in more than one cluster, potentially resulting in misclassification. Although groups differed according to characteristics not used as LCA indicators, such as intention to continue using DHTs among Adopters, membership was not validated statistically using clinical outcomes such as blood glucose values. Possible differences in individual characteristics between those recruited from Evidation vs. Facebook were not accounted for in models. Clinical characteristics such as insulin use, HbA1c in the past 3 months, and mental health conditions, were self-reported which may be subject to recall bias among others. In addition, Adopters reported a range of DHT types they have had experience with; their attitudes toward DHTs are likely influenced by differing technology reference points. Future work should also consider whether latent class membership is additionally associated with the type of DHT one uses (e.g. telehealth services vs. self-guided education). Finally, this discussion focuses primarily on personal barriers to adoption from the perspective of someone with T2D, rather than external barriers such as payor coverage or provider challenges.

## Conclusions

For adults with T2D, there is a need to examine a range of characteristics potentially associated with DHT adoption and engagement, including factors that go beyond demographic and clinical characteristics, and include attitudes toward DHTs. In this study, self-efficacy for diabetes management, health activation, perceived barriers to using DHTs, and HbA1c emerged as characteristics that may help identify different subgroups of Adopters and Non-Adopters. Identification of these subgroups can inform the development of adoption and continued user engagement strategies, such as provision of technological support to use DHTs and comprehensive support for mental and physical comorbidities, that are needed to fully realize the potential of DHTs for heterogeneous users. These findings suggest that in T2D, a tailored approach is required for promoting understanding and adoption of DHTs among Non-Adopters and continued DHT engagement among Adopters.

## Supplementary data

Supplementary material is available at *Translational Behavioral Medicine* online.

ibae034_suppl_Supplementary_Material

## Data Availability

De-identified data from this study are not publicly available.

## Welfare of Animals

This article does not contain any studies with animals performed by any of the authors.
