# Supplementary material for: Behavioral Engagement and Activation Model Study (BEAMS): A latent class analysis of adopters and non-adopters of digital health technologies among people with Type 2 diabetes
Source: Transl Behav Med. 2024 Jul 2;14(8):491–8. doi: 10.1093/tbm/ibae034 (PMC11282572; doi:10.1093/tbm/ibae034)
Supplement: ibae034_suppl_Supplementary_Material [file ibae034_suppl_supplementary_material.docx]

SUPPLEMENTARY TABLES

Table 1: Latent Class Analysis indicator variable descriptions

| Indicator Variables  (yes = 1) | Applied to cohort | Descriptions | Response Options |
| --- | --- | --- | --- |
| Has technology barriers | Both | Has at least one technological barrier to using DHTs to manage diabetes | “Too much time to use or setup”, “unsure where to find the technology” |
| Needs support to use tech | Both | Needs additional support to use DHTs | “Audio recordings”, “larger fonts”, “color choices”, “training on how to use the technology” |
| Has concerns with technology | Both | Has at least one concern with using DHTs to manage diabetes | “Negative past experience with technology”, “data privacy and security concerns”, “too expensive”, “other health problems”, “would not help me”, “could achieve goals without it”, “found other ways to manage”, “not enough motivation” |
| Motivated by social influence | Both | Would be motivated to use DHTs in response to social influence | “Healthcare provider encouraged me to use a technology”, “family or friends encouraged me to use a technology” |
| Motivated by daily activities | Both | Would be motivated to use DHTs for daily activities | “Help me return to doing social activities”, “be more productive at work” |
| Motivated by improving health | Both | Would be motivated to use DHTs for health improvement | “Help me feel better overall”, “help me with long-term management”, “prevent diabetes worsening”, “increase my life expectancy”, “achieve health goals” |
| Knows about digital health technology | Both | Knows about DHTs for diabetes management | “Knows about digital health technologies” |
| Trusts in data accuracy | Both | Trusts in the accuracy of health- related apps | “Very much trust”, “moderately trust” |
| Has mental health condition | Both | Reports depression, anxiety, or other mental health condition | “Depression”, “Anxiety”, “Other mental health conditions” |
| Reports elevated HbA1c | Both | Self-reports an HbA1c in the past 3 months above 6.5 | “Between 6.6 and 7.5”, “Greater than 7.5” |
| Currently taking insulin | Both | Uses any kind of insulin | “Premixed Insulin”, “Short-acting Insulin”, “Basal Insulin”, “Fixed ratio combination of an GLP-1RA and basal insulin” |
| High diabetes self- efficacy | Both | Confident about self-managing their diabetes | >=70 score on “Self-efficacy for diabetes” (SED) |
| High health activation | Both | Is engaged with their overall healthcare | >=80 score on “Consumer Health Activation Index” (CHAI |
| Was not adherent with technology | Adopters  only | Had not used digital health technologies for enough time | “used them for enough time to improve your diabetes  or for the length of time your healthcare provider recommends” |
| Had positive past outcome | Adopters only | Reports improvement in health from past use of technology | “Just a little bit improved”, “Somewhat improved”, “Very much improved” |
